# Exploring how arm movement moderates the effect of lower limb muscle fatigue on dynamic balance in healthy youth

**DOI:** 10.3389/fspor.2024.1391868

**Published:** 2024-05-23

**Authors:** Katharina Borgmann, Jendrik Ferdenhert, Alexandra C. Neyses, Julian Bauer, Mathew W. Hill, Thomas Muehlbauer

**Affiliations:** ^1^ Division of Movement and Training Sciences/Biomechanics of Sport, University of Duisburg-Essen, Essen, Germany; ^2^ Department of Sport Science, Human Performance Research Centre, University of Konstanz, Konstanz, Germany; ^3^ Centre for Physical Activity, Sport and Exercise Sciences, Coventry University, Coventry, United Kingdom

**Keywords:** postural control, upper body strategy, arm position, lower extremities, reaching movement, exhaustion, youth

## Abstract

**Background:**

In young adults, there is evidence that free arm movements do not help to compensate muscle fatigue-induced deteriorations in dynamic balance performance. However, the postural control system in youth is immature, and as a result, the use of arm movements may provide a compensatory “upper body strategy” to correct fatigue-related balance impairments. Thus, the purpose of the present study was to compare the effects of free vs. restricted arm movement on dynamic balance performance prior and following exercise-induced muscle fatigue.

**Methods:**

Forty-three healthy youth (19 females; mean age: 12.8 ± 1.9 years) performed the Y Balance Test–Lower Quarter before and immediately after a fatiguing exercise (i.e., repetitive vertical bipedal box jumps until failure) using two different arm positions: free (move the arms freely) and restricted (keep the arms akimbo) arm movement.

**Results:**

Muscle fatigue (*p* ≤ 0.033; 0.10 ≤ *η*_p_^2^ ≤ 0.33) and restriction of arm movement (*p* ≤ 0.005; 0.17 ≤ *η*_p_^2^ ≤ 0.46) resulted in significantly deteriorated dynamic balance performance. However, the interactions between the two did not reach the level of significance (*p* ≥ 0.091; 0.01 ≤ *η*_p_^2^ ≤ 0.07).

**Conclusion:**

Our findings indicate that the use of an “upper body strategy” (i.e., free arm position) has no compensatory effect on muscle fatigue-induced dynamic balance deteriorations in healthy youth.

## 1 Introduction

The negative influence of motor performance fatigue (i.e., reversible exercise-induced reduction in neuromuscular performance) on postural control in healthy youth is firmly established (1–5). For example, Steinberg et al. (3) investigated fatigue-induced performance changes in boys (*N *= 13; mean age: 11.5 ± 1.8 years) and girls (*N *= 17; mean age: 13.8 ± 2.9 years). The authors reported that postural sway was significantly increased while standing on both legs immediately after a 20-m shuttle-run aerobic fatigue test. Moreover, Pau et al. (2) compared balance performance before and after a repeated sprint ability test (i.e., 6 repetitions of maximal 2 × 15-m shuttle sprints) in adolescents (*N *= 21; mean age: 14.5 ± 0.2 years) and showed significantly increased sway values during single and double leg stance. Lastly, Guan et al. (4) tested 13 children aged between 9 and 11 years that performed a fatigue protocol consisting of two sets of 30-s double leg kicks at the maximum frequency and consecutive frog jumps until exhaustion. After finishing the protocol, reach distances for the Y Balance Test–Lower Quarter (YBT–LQ) were significantly reduced.

At the same time, there is growing evidence that arm movements can contribute to stabilise balance in children and adolescents (6, 7). For instance, Hill et al. (6) investigated 29 children (mean age: 10.6 ± 0.5 years) that completed several dynamic balance assessments with free and restricted arm movement. The free use of arm movement resulted in significantly larger YBT–LQ reach distances and shorter durations for the timed balance beam walking test. Further, Muehlbauer et al. (7) studied 40 children (mean age: 11.5 ± 0.6 years) and 30 adolescents (mean age: 14.0 ± 1.1 years) that performed static and dynamic balance tests under free vs. restricted arm movement conditions. The results showed better performance values for the single leg stance test, the YBT–LQ, and the 3-m beam walking backward test when participants were instructed to move their arms freely instead of to claps their hands in front of the body at the waist during the balance tasks.

Although the benefit of free arm movement on postural control is well known, the possibility of a compensatory effect on muscle fatigue-induced impairments in balance performance has hardly been investigated so far. To date, there is only one study^1^ from our lab that examined the role of arm movements during dynamic balance testing before and after lower limb muscle fatigue. Specifically, healthy young adults (*N *= 52; mean age = 22.6 ± 1.6 years) performed the YBT–LQ with free and restricted arm movements before and immediately after a fatiguing exercise (i.e., repetitive vertical bipedal box jumps until failure). We found that restriction of arm movement and application of fatigue independently, but not the interaction between the two, resulted in significantly deteriorated lower limb reach distances. Accordingly, in young adults, free arm movement does not seem to compensate for muscle fatigue-induced dynamic balance deterioration. However, a direct transfer of these findings to healthy youth is not possible because the maturation of postural control mechanisms is still incomplete (8–10). Specifically, deficits in static and dynamic balance performance are evident in children and adolescents when compared to young adults (11, 12), indicating different strategies for balance control (8, 13). Therefore, free arm movements may have a significant role in compensating for muscle fatigue-induced performance deteriorations in dynamic balance in youth. Thus, exploring how arm movement moderates the effect of lower limb muscle fatigue on measures of dynamic balance in healthy children and adolescents will enable us to better understand how the upper body is used to control posture in youth. From a practical perspective, the present study could add insights on how to effectively design balance training programmes. Precisely, allowing free arm movements would be a relatively simple task manipulation that could be used in long-lasting programmes to continue balance training despite exercise-induced muscle fatigue.

The current study explored how arm movement moderates the effect of lower limb muscle fatigue on dynamic balance in healthy youth. To reject or confirm previously reported effects of muscle fatigue on dynamic balance performance in healthy young adults^1^, we applied the same methodology in terms of experimental procedure, fatigue protocol, and dynamic balance assessment. Based on previous work (4, 6), we hypothesised that lower limb muscle fatigue and restricted arm movement would lead to impaired dynamic balance performance, but performance impairment when fatigued would be less evident when the participants are allowed to use their arms for postural control during balance testing.

## 2 Material and methods

### 2.1 Participants and sample size estimation

Forty-three healthy, physically active subjects voluntarily participated in the present study. Their characteristics are shown in Table 1. With the help of G*Power 3.1.9.8 (14), an *a priori* power analysis (*f *= 0.25, *α *= 0.05, 1-*β *= 0.80, number of groups: *n *= 1, number of measurements: *n *= 4) was performed for measures of dynamic balance performance (15, 16). The analysis revealed that *N *= 41 participants would be sufficient to find statistically significant repeated measures analysis of variance (ANOVA) effects. The participants were recruited via an information event from public primary and secondary schools in the Ruhr area of North Rhine-Westphalia, Germany. Inclusion criteria were willingness to participate and age 10–16 years. We excluded participants suffering from any problems that may interact with postural control including musculoskeletal dysfunction, neurological impairment, orthopaedic pathology, or an injury during the last three months. Participant's assent and written informed consent of the parents or legal guardians were obtained before the start of the study. The Human Ethics Committee at the University of Duisburg-Essen, Faculty of Educational Sciences approved the study protocol.

**Table 1:** Participant characteristics.

Characteristic	Youth (*n *= 43)
Sex (females; *n*)	19
Mean age (years)	12.8 ± 1.9
Age range (years)	10–16
Body height (cm)	159.7 ± 10.2
Body mass (kg)	52.8 ± 12.5
Body mass index (kg/m^2^)	19.6 ± 5.5
Leg length (cm)	89.3 ± 7.2

Values are means ± standard deviations.

### 2.2 Experimental procedure

**Figure 1:**
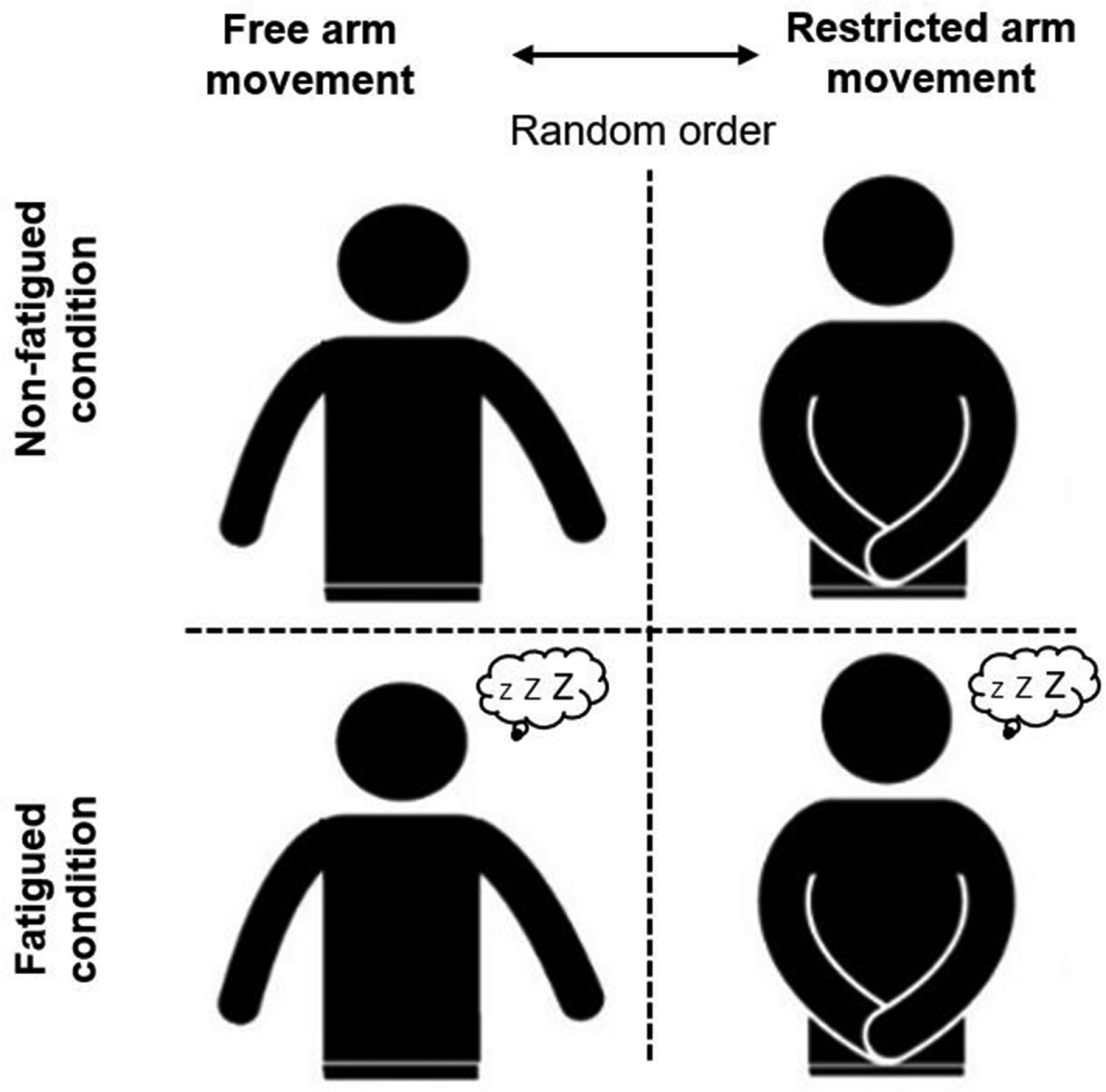
Schematic diagram of the different experimental conditions.

A single-group repeated-measures design that included two sessions separated by one week was used to assess the effects of exercise-induced lower limb muscle fatigue on measures of dynamic balance performance (Figure 1). At the beginning of each testing session, participants received instructions on the specific procedure. Afterwards, a standardised warm-up protocol was conducted that consisted of three minutes of rope skipping, two minutes of active stretching exercises for the lower body (i.e., calf muscles, quadriceps muscles, hamstring muscles, hip muscles), and two minutes of a familiarisation phase with submaximal single leg reaching movements. Thereafter, the participants executed the YBT–LQ that was followed by a rating of their initial perceived exertion. Afterwards, they performed the fatigue protocol, followed by another rating of their perceived execution. Immediately afterwards, the YBT–LQ was carried out again and the experimental procedure ended. The same procedure was repeated one week later. The permission to use (free) or not to use (restricted) arm movements while performing the single leg reaching movements prior and following the fatigue protocol was randomised between participants to avoid potential bias. Precisely, the free source Research Randomizer (www.randomizer.org) was used to randomly assign the participants to the experimental conditions. For the free arm movement condition, participants were instructed to move their arms freely and to their advantage. For the restricted arm movement condition, participants were asked to keep their arms akimbo and compliance was visually monitored. The experimental procedure described above was carried out in the morning (between 9 and 11 a.m.) at a room temperature of approx. 20°C in the gym hall of the respective school.

### 2.3 Anthropometric measurements

The participants were asked to stand straight and upright without shoes while their body height was measured to the nearest 0.1 cm using a Seca 217 linear measurement scale (Seca, Basel, Switzerland). Participants wore light sportswear but no shoes when their body mass was measured to the nearest 100 g using a Seca 803 electronic scale (Seca, Basel, Switzerland). The body mass index was calculated by dividing the body mass by the body height squared (kg/m^2^).

### 2.4 Fatigue protocol

Lower limb muscle fatigue was individually induced by repeated vertical bipedal box jumps. Participants performed as many metronome-paced (70 beats per minute) box jumps (box height: 28 cm) as possible until failure (17). Failure was defined as the time when the participants could no longer follow the pace of the metronome. The number of repetitions in the first set represented the reference (i.e., 100%) for the following set. During this set, participants were again instructed to perform as many repetitions as possible until failure. If at least 60% of the first set was achieved (18), another set followed, otherwise the fatigue protocol ended. The rest period between sets amounted to one minute. The number of sets as well as repetitions per set was manually recorded.

### 2.5 Dynamic balance assessment

The YBT–LQ was administered using the Y Balance Test Kit (Functional Movement Systems, Chatham, USA). The device consists of a central standing platform on which three tubes are attached in different directions. These indicate the reach directions anterior (AT), posteromedial (PM), and posterolateral (PL) and are marked in 1.0 cm increments for measurement purposes. The three tubes were equipped with a movable reach indicator. Participants were instructed to stand without shoes on the central platform with their dominant leg. To determine the dominant leg, the participants were asked, “Which foot do you use to kick a ball?”. In three practice trials, followed by three data collection trials for each reach direction, participants were asked to use their free leg to push the reach indicator as far as possible in the AT, PM, and PL directions. A trial was discarded and repeated if a participant (a) failed to maintain the one-legged stance (i.e., touched the ground with the reach leg), (b) raised the supporting leg from the central platform, (c) used the reach indicator for support of body weight, (d) failed to maintain contact with the reach indicator at the most distal point, (e) failed to return the reach leg to the centre of the central platform, or (f) released the arms from the hips with limited arm movement has. The trial with the greatest reach distance (measured in cm) per reach direction was used for the subsequent analyses. More specifically, the distance per reach direction was normalized [% leg length (LL)] by dividing the maximum reach distance (measured in cm) by the dominant lower limb length (measured in cm) and multiplying by 100. Additionally, the composite score (CS) was calculated (%LL) (19). This represents the sum of the maximum range (cm) per range direction divided by three times LL (cm). The result is then multiplied by 100. Lower limb length (measured in cm) was recorded from the anterior superior iliac spine to the most distal part of the medial malleolus (20). The YBT–LQ is a valid (AUC-values: ≥74%) and reliable (ICC-values: 0.40–0.96) tool to assess dynamic balance performance in children and adolescents (21, 22).

### 2.6 Rating of perceived exertion

We used a 6–20 Borg scale (23) to assess the level of subjectively perceived and verbally expressed exertion prior and following the fatigue protocol with 6 indicating “no exertion at all” at all and 20 indicating “maximal exertion”.

### 2.7 Statistical analyses

Descriptive statistics were presented as group mean value ± standard deviation (*SD*). Before conducting inference statistics, normal distribution (Shapiro–Wilk Test) and variance homogeneity (Mauchly Test) were checked and confirmed. In terms of dynamic balance performance, series of 2 (fatigue level: non-fatigued, fatigued) × 2 (arm movement: free, restricted) repeated measures ANOVA were performed. Regarding perceived exertion, the Wilcoxon signed rank test was used to detect differences prior vs. immediately after the fatigue protocol. The significance level was *a priori* set at *α* < 0.05. For the repeated measures ANOVA, partial eta-squared (*η*_p_^2^) was calculated and reported as small (0.02 ≤ *η*_p_^2^ ≤ 0.12), medium (0.13 ≤ *η*_p_^2^ ≤ 0.25), or large (*η*_p_^2^ ≥ 0.26) (24). All analyses were performed using SPSS version 28.0 (IBM Inc., Chicago, IL).

## 3 Results

### 3.1 Trial-by-trial reliability

Irrespective of test condition and reach direction, trial-by-trial reliability was predominately “excellent” (i.e., ICC > 0.75) (data not shown).

### 3.2 Rating of perceived exertion

Participants performed between 2 and 5 sets of bipedal box jumps until failure, with an average jump number of 29.8 ± 16.9, 25.6 ± 16.1, 23.9 ± 14.2, and 21.0 ± 9.6 for the second, third, fourth, and fifth set, respectively. As a result, we detected significantly increased levels of perceived exertion in both arm movement conditions from “very light exertion” to “hard exertion” (free: non-fatigued = 8.9 ± 2.6; fatigued = 14.8 ± 2.4; *Z* = −5.735, *p* < 0.001; restricted: non-fatigued = 8.8 ± 2.2, fatigued = 15.1 ± 2.2; *Z* = −5.724, *p* < 0.001).

### 3.3 Dynamic balance performance

**Figure 2:**
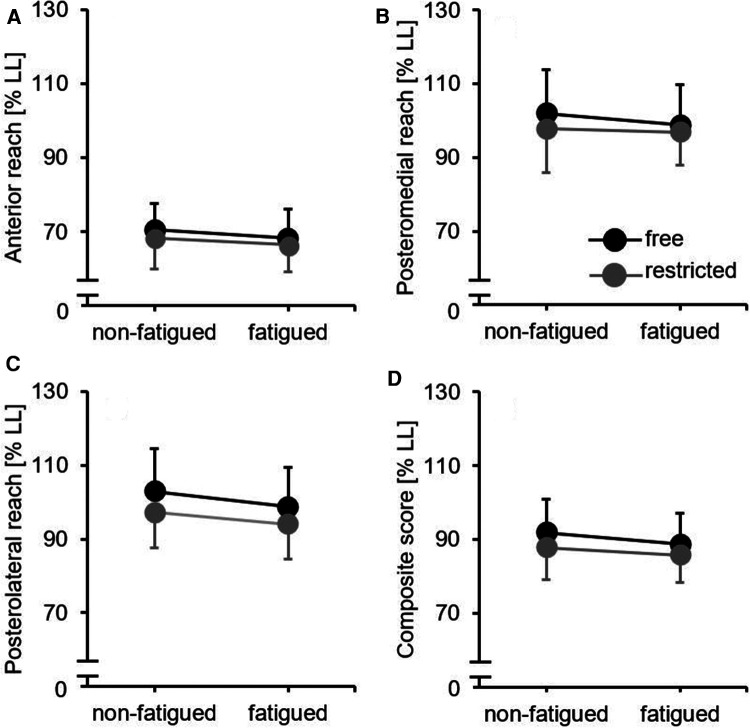
Dynamic balance performance by fatigue (non-fatigued vs. fatigued) and arm movement (free vs. restricted) conditions for (**A**) anterior reach direction, (**B**) posteromedial reach direction, (**C**) posterolateral reach direction, and (**D**) composite score. Black filled circles mean free arm movement condition and grey filled circles mean restricted arm movement condition. LL, leg length.

The results of the descriptive and inference statistics are shown in Table 2. We detected significant main effects of fatigue (*p* ≤ 0.033; 0.10 ≤ *η*_p_^2^ ≤ 0.33) and arm movement (*p* ≤ 0.005; 0.17 ≤ *η*_p_^2^ ≤ 0.46), indicating poorer dynamic balance performances in the fatigued compared to the non-fatigued condition and for the restricted compared to the free arm movement position (Figures 2A–D). However, we did not detect significant fatigue × arm movement interactions.

**Table 2:** Descriptive and inference statistics of dynamic balance performance by fatigue (non-fatigued vs. fatigued) and arm movement (free vs. restricted) conditions.

Outcome	Non-fatigued		Fatigued		Main effect: fatigue	Main effect: arm movement	Interaction effect: fatigue × arm movement
	Free	Restricted	Free	Restricted	*p*-value (*η*_p_^2^)		
AT (% LL)	70.5 ± 7.1	68.2 ± 8.3	68.4 ± 7.7	66.5 ± 7.4	**.004** **(.18)**	**.005** **(.17)**	.688 (.01)
PM (% LL)	102.0 ± 11.9	97.7 ± 11.7	98.8 ± 11.0	96.9 ± 9.1	**.033** **(.10)**	**<.001** **(.40)**	.091 (.07)
PL (% LL)	102.8 ± 11.6	97.3 ± 9.8	98.8 ± 10.7	94.0 ± 9.4	**<.001** **(.29)**	**<.001** **(.43)**	.609 (.01)
CS (% LL)	91.8 ± 9.1	87.7 ± 8.7	88.7 ± 8.5	85.8 ± 7.4	**<.001** **(.33)**	**<.001** **(.46)**	.129 (.05)

Bold values indicate statistically significant main effects (*p* < .05). Threshold values for the *η*_p_^2^-value were.02 ≤ *η*_p_^2^ ≤ .12 = small, .13 ≤ *η*_p_^2^ ≤ .25 = medium, and *η*_p_^2^ ≥ .26 = large. AT = anterior; CS, composite score; LL, leg length; PM, posteromedial; PL, posterolateral.

## 4 Discussion

The purpose of this study was to explore the effect of lower limb muscle fatigue on dynamic balance performance in free and restricted arm movement conditions in healthy youth. Our investigation builds on a recent study^1^ with the following major findings: (a) in line with the first part our hypothesis, we detected deteriorations in dynamic balance performance under fatigued compared to non-fatigued conditions and with restricted compared to free arm movements; (b) contrary to the second part of our hypothesis, we did not observe a compensatory effect of free arm movements on muscle fatigue-induced impairments in dynamic balance performance (i.e., no fatigue by arm movement interaction).

### 4.1 Influence of lower limb muscle fatigue on dynamic balance performance

As expected, and in agreement with previous literature in youth (4) motor performance fatigue resulted in significantly reduced lower limb reach distances and the CS under restricted and free arm movement conditions. In this regard, Guan et al. (4) compared lower limb reach distances before and after exercised-induced muscle fatigue in children (mean age: 9.9 ± 0.8 years) and observed significantly degraded lower limb reach distances (except for the PL reach direction) and the CS. The impaired performance in dynamic balance after the fatigue protocol can be explained, among other things, by the fact that metabolic changes like the accumulation of lactic acid led to a desensitization of the muscle spindles (25). Additionally, these metabolic by-products may have been distributed to remote muscles, decreasing their voluntary activation (5) and hindering their contractile ability (26). In this context, Alderman (27) stated that a high level of fatigue can impair the accuracy of neuromuscular coordination tasks.

### 4.2 Contribution of arm movement to control dynamic balance

As predicted, and consistent with previous studies (6, 7), restricting arm movements resulted in significantly impaired lower limb reach distances and the CS under non-fatigued and fatigued conditions. In this context, Hill et al. (6) examined the effects of arm movements on the performance of dynamic postural tasks in 14 healthy boys and 10 girls. Restricting arm movements elicited significant deteriorations in YBT–LQ reach distances and 2-m tandem walk time on a balance beam. Furthermore, Muehlbauer et al. (7) explored the role of arm movements during dynamic balance tasks in children (*n *= 40) and adolescents (*n *= 30). Again, the restriction compared to the free use of arm movements resulted in smaller YBT–LQ reach distances and less steps while walking backward. Different mechanisms can be attributed to explain the deteriorations in dynamic balance during restricted arm movement conditions. Firstly, the arms cannot be used as a counterweight to shift the body centre of mass away from the direction of instability (28). Secondly, restoring torques to reduce angular momentum of the body (29) cannot be generated. Thirdly, the arms cannot be applied to increase the moment of inertia (6).

### 4.3 Compensatory effect of arm movement on fatigue-induced impairments in dynamic balance

Additionally, we further assumed that the fatigue-induced decrements in dynamic balance would be lowered when the participants are allowed to use their arms for postural control. However, we did not detect a fatigue by arm movement interaction. This result is contrary to our hypothesis but confirms the findings of our previous study in young adults^1^. In sum, this indicates that the use of an “upper body strategy” has no compensatory effect on fatigue-induced dynamic balance impairments in young adults as well as in youth. Therefore, practitioners are advised to deal with fatigue-related dynamic balance impairments by providing a rest period rather than instructions on the use of free arm movement. In this regard, Johnston et al. (15) applied the YBT–LQ before and after (0, 10, and 20 min) a fatigue protocol (i.e., modified 60-s Wingate protocol). They found that the AT reach direction returned to pre-fatigue level within 10 min and the PM reach direction within 20 min, while the PL reach direction did not return within this time. Future studies could examine whether these rest periods also apply to our fatigue protocol (i.e., repetitive vertical bipedal box jumps until failure) and can be reduced by using arm movements.

One possible reason for the absence of a fatigue by arm movement interaction could be that the YBT–LQ requires not only balance but also lower-extremity muscle strength (30), flexibility (31), and core control (32). Therefore, these additional factors could have contributed that the assumed compensatory effect of arm movement on fatigue-induced impairments in dynamic balance was not evident.

### 4.4 Limitations

The present study has some limitations. Firstly, we only measured behavioural data (i.e., reach distances) but no kinematic (e.g., joint angles) or electromyographical (i.e., muscle activation) data, which limits our understanding about the role of arm movements on postural control following lower limb muscle fatigue. Secondly, muscle fatigue was only assessed subjectively (i.e., 6–20 Borg scale) but not objectively (e.g., blood lactate). Thirdly, our study was limited to healthy youth and the findings cannot be directly generalized to older adults whose neuromuscular system is influenced by ageing processes, which may make them more dependent on arm movements for postural control following lower limb muscle fatigue.

## 5 Conclusions

The result of performance impairments following exercise-induced lower limb muscle fatigue and while performing the dynamic balance task with restricted arm movements but not the combination of both factors indicates that the “upper body strategy” (i.e., free arm position) has no compensatory effect in healthy youth. Therefore, teachers and coaches are advised to provide sufficient rest periods for neuromuscular recovery rather than to ask young individuals to use their arms freely when the goal is to compensate lower limb muscle fatigue-induced deteriorations in dynamic balance performance.

## Data Availability

The raw data supporting the conclusions of this article will be made available by the authors, without undue reservation.

## Footnotes

^1^Borgmann K, Brinkmann R, Bauer J, Hill MW, Muehlbauer T. Effect of lower limb muscle fatigue on dynamic balance performance in healthy young adults: role of arm movement. *Sci Rep*. (2024).
